# Progress in Cancer

**Published:** 1900-11-24

**Authors:** 


### Progress in Cancer.

**Pathology.**—Adami,[6] in an able paper on "Cell Differentiation and Proliferative Capacity," points out that where cells are undergoing active division for the purposes of growth, replacing of waste, or reparation for injury, the "mother cells" revert to a more or less embryonic type.

Thus in skin the Malpighian layer is the closest approximation to the embryonic type, and comprises the "mother cells" of the tissue. If by reason of injury the prickle cells undergo mitosis they lose their "prickles," and thus revert to a simpler or more embryonic type.

In bone, production of bone tissue is not due to pre-existing bone cells, but to osteoblastic tissue, lying in immediate connection with the vessels of the inner layer of periosteum, or again coursing through Haversian canals and between the laminæ. These on injury apply themselves to the pre-existing bone, and, multiplying, deposit a calcareous matrix which gradually assumes the character of true bone. In testicular, ovarian, mammary epithelium and columnar and ciliary epithelia of mucous membranes there are mother-cells lying at the bases of the fully developed cells.

After adducing similar facts from the development of lymph glands, peritoneum, muscle, &c., the writer lays down the following laws:—

1. That the fully differentiated cells of a tissue proper never arise from cells that are themselves fully differentiated.

2. Under the *normal* conditions of growth and during physiological regeneration, the fully differentiated cells would seem in nearly every case to be developed from "mother cells"—undifferentiated or but partially differentiated cells—which, themselves, throughout the term of life of the individual never attain to the full differentiation peculiar to the tissue to which they belong, and which indeed they produce. For these mother-cells by division give origin to the daughter cells, and it is the daughter cells which attain full differentiation and form the specific cells of the tissue. More rarely the functional cells themselves, by reversion to a more embryonal type, can take on the properties of mother-cells.

3. Under *abnormal* conditions the fully differentiated functioning cells of certain tissues are capable of proliferation and giving rise to cells of like nature, but this is only after a preliminary reversion to a simpler, more embryonic type. The fully differentiated cell as such is incapable of proliferation.

4. Or, otherwise, the energy stored up by the cell may be expended in one or two directions, but not in both, either in functional activity or in preparation for proliferation; and, the structure of a cell being the expression of the activity of that cell, the expenditure of energy in either direction is attended by corresponding morphological or structural differences in the cell.

5. The more highly differentiated a cell, the more highly elaborated its structure, the less the ease with which it reverts, and the less the liability to reversion to a simpler reproductive form; the simpler the cell, the greater the ease and the greater the liability to such reversion. It is thus possible, at the one extreme, to conceive cells so simple in function and in structure that functional or reproductive activity may be called into play indifferently without *recognisable* preliminary structural alteration, and at the other cells so highly differentiated that the capacity for proliferation has become entirely lost.

In speaking of metaplasia the writer holds that the law enunciated explains how a columnar can become converted into a squamous epithelium, and one form of connective tissue into another; for they come from the same mother-cell under the influence of different environment. The reverse of the law, that the further the daughter-cells depart from the maternal type the less their proliferative capacity, would seem true, namely, that the more the daughter-cells in a given tissue retain the characteristics of the mother-cells the greater the proliferative capacity.

He does not hold that this is more than "one important principle" in the development of tumours proper; but apart from the discussion of the essential stimulus necessary for a tumour, he holds that it explains why tumours, where tissue cells are more or less faithfully reproduced, are essentially *benign*, while malignancy is characterised by tumorous formation of a pronounced embryonic type.

Such proliferative cells then are considered as being derived from—

1. Embryonic cell "*rests*," which suddenly proliferate.

2. *Mother-cells* of a tissue which, remaining undifferentiated, but capable of active proliferation throughout life, now assume excessive proliferative powers, the daughter-cells retaining more or less the character and properties of mother-cells.

3. Differentiated cells which, reverting to a simpler more embryonic type, with this diversion gain the capacity for active and excessive proliferation.

The tendency to the development of glandular cancer in later life bears some relationship to the reversion and degeneration of gland cells at this period. The tissues become exhausted and the cells lose their higher differentiation, and revert to a simpler type, with more proliferative powers.

**The Inoculation of Cancer.**—Podwyssotzki[7] has produced tumours in rabbits and guinea-pigs by inoculating small pieces of cabbage infected by the parasite "Plasmodiophora Brassica." The tumours were not cancerous in nature. The parasites were almost invisible in the protoplasm of the tumour cell, and hence the author warns against too hasty a conclusion against the parasitic theory of cancer, through the inability to discover "conspicuous and easily-demonstrated parasitic organisms in cancer."

Sjobring[8] has succeeded in isolating organisms "resembling the rhizopods" from about thirty different tumours of carcinomatous, sarcomatous, or myomatous types. He has incubated them on a special medium—viz., 8 per cent. peptone gelatine and 1·5 per cent. concentrated aqueous solution of potash soap, made of human fat, 1 per cent. cane or grape sugar, and 5 per cent. sterile ascitic fluid, the whole rendered sterile. He describes six various forms of these organisms, including three chief varieties: First, amœboid; second, rhizopods; third, involution or resting forms. He has obtained four positive results on inoculating eight white mice, and producing lesions more or less characteristic of the primary tumours. The parasites were again found in these tumours.

Joseph Sailer[9] writes an article on the inoculability of cancer: he reviews the work reported, positive and negative in its results on the implantation of malignant tissue into men and animals. The negative results, he holds, far outweigh the positive. The inoculation of the lower animals is, in his opinion, quite indecisive, for he says the results are invariably doubtful. Not a single positive experiment has been carried out with precautions regarding the histological nature of the growth and its development in the lower animals that would render it worthy of a moment's consideration. There is no reason to believe that cancer tissue acts in any respect differently from any other tissue introduced into the lower animals. It produces the same effects and suffers the same changes. For further investigation of the subject he urges the inoculation of the lower animals with tumour-tissue from lower animals and not from man. Also a more exact knowledge is required of the blastomycetes from a botanical standpoint, and a more careful description of the lesions produced.

**The Cancri Amœba.**—Gustav Eisen[10] gives a preliminary report upon his work upon the parasitic amœbæ found in epithelial carcinomata.

He urges the necessity of fixing the "cancriamœba" so instantaneously that no contraction can take place. Formerly, owing to this contraction, the parasite had been mistaken for cell-degeneration or cornifications, &c.

He finds that cold contracts the amœbæ, and so he warms the fixative, and uses for this medium a solution containing—

| | |
|---|---|
| Bichromate of potash . . | 8 parts |
| Glacial acetic acid . . . | 5 „ |
| Water . . . . . . . | 100 „ |

using twenty times as much medium as tissue, and changing as often as cloudiness appears. Small pieces of tumour are cut out of the mass ($\frac{1}{8}$ to $\frac{1}{2}$ in. in diameter), and while yet warm are dropped into the medium at the temperature of the blood. Then the preparation is kept in running water until the colour is gone (*circa* 12 hours). It is then taken through the alcohols to bergamot oil (twice), xylol (twice), into bergamot again to prevent too violent evaporation of xylol in paraffin. When perfectly clear the sections are cut. The process will have taken about three days. The sections must not be thicker than 4 $\mu$. If thicker the stain does not obtain fair play. Fixed on slide by alcohol method, and the paraffin being removed, the section is stained with 1 per cent. Grübler's eosin in alcohol, being left all night (alcohol removed by water) and counterstained with 1 per cent. methylene blue "O" in water and 10 per cent. alcohol. Wash out stain with absolute alcohol, drop into bergamot oil free of alcohol through xylol to gum—thus in xylol (or failing this Canada balsam in xylol).

The tissue is pale red with bluish nuclei, the amœbæ intensely red with deep blue nuclei.

"Cancriamœba macroglossa," 7 to 30 $\mu$, extrudes a tongue-like process and enters epithelial cells, devouring the contents and eventually lying in the vacuole thus caused. The pseudopodium acts as a locomotive organ and as a suctorial one. Several cells may be attacked at the same time by different pseudopodia of the same amœba. Sporulation probably occurs by division of the nucleus. Plimmer's organisms are probably the spores. Amitotic division also occurs.

He describes the epithelial proliferation as an attempt to enclose the parasites by means of the cells, which are best able to deal with them. Cell-nests are produced by the enclosure of the parasites, which are found in the centre, sometimes endeavouring to pierce the invading wall, sometimes dead and solidified. The presence of pseudopodia have been found actually invading cells, and the fact that the amœbæ sporulate exclude the possibility that they are blastomycetes or other fungi.

The sensitiveness of the parasites to cold suggests the treatment of carcinomata by freezing.

**Statistics.**—Mahler[11] publishes the impressions of 161 cases of mammary carcinomata. As to ætiology, he finds no positive support in the influence of the menopause, number of births, lactation, and trauma; but insterstitial mastitis he considers to play an ætiological part.

Most of the tumours were mixed forms of scirrhus and medullary cancer: 16·4 per cent. of the cases presented no swelling of glands.

| | | | |
|---|---|---|---|
| The axillary glands were affected | | 127 | times |
| supra-clavicular | „ | „ 18 | „ |
| infra-clavicular | „ | „ 5 | „ |
| cervical | „ | „ 1 | „ |
| opposite axillary | „ | „ 3 | „ |

At the operations since 1890, most of the most superficial layer of the Pectoralis major was removed; but never the whole breast muscles.

Of 133 cases operated on—

| | | |
|---|---|---|
| 21 | per cent. remain well after 3 years | |
| 14·1 | „ „ „ „ 5 „ | |
| 2 = 1·15 | „ are dead. | |

Seventy-one recurrences were met with, and 30 metastases in inner organs.

**Treatment.**—Petrini-Galatz,[12] of Bucharetz, communicates the case of a man of 40 years, who suffered from multiple tumours, occupying the fauces, the palatine arch, the gums, the forehead, the neck, &c. Each of these tumours was of the size of a small chestnut, and presented a semi-hard consistency. They were situated under the skin, to which they were partly adherent, or infiltrated the deep tissues considerably, in the buccal cavity for instance. Histological examination of a portion cut out showed that it consisted of an embryonic vascular sarcoma. The patient was treated with "cacodylate of soda," given in intramuscular injections of about 0·05 centigr., then 0·08, 0·1, 0·12 centigr. per diem. These injections were administered partly into the buttocks, partly into the tumours of the subhyoid region; after fifty injections the resolution of the tumours was almost completely produced, and the patient enjoyed very good health. The result seems to be that non-melanotic multiple sarcomata, if treated early, are amenable to treatment with arsenic by injection.

**Cases.**—A case of epithelial cancer of bone following a fracture is reported from Maier's clinic.[13] It occurred in a girl aged 20, who in 1896 injured her ulna, the injury causing considerable pain at the time. In 1897 a thickening was noticed, and a skiagram showed a separation of the epiphysis, with a clear intervening space. Diagnosis was made of mytloid sarcoma. The ulna was resected and ivory implanted; three years afterwards the union was found strong. The preparation showed in the centre of the bone a typical epithelial cancer with marked cornification. The writer explained the case by supposing that the injury carried a deep process of epithelium downwards between the portions of fractured bone.

Newbolt[14] reports the case of a woman who had a duct carcinoma in the left breast coincidently with a glandular carcinoma in the right.

Rolleston[15] brings forward a case of primary carcinoma of the vermiform appendix.

[6] Med. Chron., June, 1900. [7] Med. Chron., June, 1900. [8] Brit. Med. Jour., Mar. 31, 1900. [9] Amer. Jour. Med. Sci., Aug. 1900. [10] Med. Rec., July 7, 1900. [11] Cent. für Chir., June 23, 1900. [12] La Sem. Méd., Aug. 15, 1900. [13] Cent. für Chir., June 23, 1900. [14] Lancet, May 26, 1900. [15] Lancet July 7, 1900.

